# Application of extracellular lipopeptide biosurfactant produced by endophytic *Bacillus subtilis* K1 isolated from aerial roots of banyan (*Ficus benghalensis*) in microbially enhanced oil recovery (MEOR)

**DOI:** 10.1007/s13205-013-0119-3

**Published:** 2013-02-12

**Authors:** Khyati V. Pathak, Hareshkumar Keharia

**Affiliations:** BRD School of Biosciences, Sardar Patel Maidan, Satellite Campus, Sardar Patel University, Vadtal Road, P.O. Box 39, Vallabh Vidyangar, 388120 Gujarat India

**Keywords:** *Bacillus subtilis*, Lipopeptide biosurfactants, Emulsifying activity, Critical micelle concentration, MEOR

## Abstract

*Bacillus subtilis* K1 isolated from aerial roots of banyan tree secreted mixture of surfactins, iturins and fengycins with high degree of heterogeneity. The extracellular extract consisting of mixture of these cyclic lipopeptides exhibited very good emulsification activity as well as excellent emulsion stability. The culture accumulated maximum surfactant up to 48 h of growth during batch fermentation in Luria broth. The emulsion of hexane, heptane and octane prepared using 48-h-old culture supernatant of *B. subtilis* K1 remained stable up to 2 days while emulsion of four stroke engine oil remained stable for more than a year. The critical micelle concentration of crude lipopeptide biosurfactant extracted by acid precipitation from 48-h-old fermentation broth of *B. subtilis* K1 was found to be 20.5 μg/mL. The biosurfactant activity was found to be stable at 100 °C for 2 h, over a pH range of 6–12 h and over an NaCl concentration up to 10 % (w/v). The application of biosurfactant on laboratory scale sand pack column saturated with four stroke engine oil resulted in ~43 % enhanced oil recovery, suggesting its suitability in microbially enhanced oil recovery.

## Introduction

Current worldwide market for surfactants is around $9.4 billion per annum. Almost all surfactants currently in use are chemically synthesized (Desai and Banat 1997; Mukherjee 2007). Biosurfactants are surface active amphiphilic molecules synthesized by microorganisms. These surface active molecules reduce surface tension as well as interfacial tension in aqueous solutions and hydrophobic mixtures (Banat 1995). Most microbial surfactants are complex structurally diverse groups of molecules comprising cyclic lipopeptides, glycolipids, phospholipids, fatty acids and polysaccharide protein complexes (Cooper and Goldenberg 1987; Rosenberg 1986; Desai and Banat 1997). The important features of biosurfactants over chemically synthesized surfactants are their biodegradability, bioavailability, lower toxicity, higher foaming, and high specific activity at extreme pH, temperature and salinity (Desai and Banat 1997; Rosenberg 1986). Biosurfactants owing to their unique properties are being increasingly favored in the place of chemically synthesized surfactants for industrial applications such as emulsifiers in food and pharmaceutical industries, as surfactants in laundry, as biological control agents, in mobilization of heavy oil spills, oil-pollution control, cleaning of oil sludge from storage facilities, bioremediation of oil-contaminated soil and enhanced oil recovery (EOR) (Banat et al. 1991; Kosaric 1992; Banat 1995; Nitschkea and Cosat 2007; Mukherjee 2007; Rahman and Gakpe 2008; Brown 2010). The left over oil from wells upon primary and secondary recovery processes has to be recovered by specialized enhanced oil recovery techniques (EOR) (Morkes 1993; Brown 2010). The currently available chemical EOR processes are designed based on polymer flooding, surfactant flooding, alkaline flooding, and injection of steam or in situ combustion (Brown 2010). The use of chemical surfactants for EOR is undesirable as it is hazardous, costly and may leave undesirable residues, difficult to dispose off without adversely affecting the environment (Kosaric 1992; Banat 1995). The technique which uses microbes or their products to enhance oil recovery is known as microbially enhanced oil recovery (MEOR) and was first proposed by Beckman (1926). This MEOR technique has proved to be a better alternative to the currently available chemically EOR as microbes or microbial products are generally less toxic, biodegradable as well as equally effective to chemical surfactants with additional benefits of lower energy and capital requirements (Rosenberg 1986; Cooper and Goldenberg 1987; Sarkar et al. 1989; Banat 1995). The mechanisms of MEOR include: gas formation, acid production, degradation of lime stone matrices, solvent production, production of polymers, reduction in oil viscosity and interfacial tensions by microbial biosurfactants (Jack 1988; Khire and Khan 1994). Three main strategies have been developed for use of biosurfactants in MEOR viz. (1) injection of biosurfactant producing microbes along with nutrients into the oil reservoir through the reservoir rocks facilitating multiplication of microorganisms in situ, (2) biostimulation of indigenous biosurfactant producing microorganisms by injection of selected nutrient into oil reservoir to stimulate the microbial growth and (3) injection of ex situ produced biosurfactants into reservoir (Shennan and Levi 1987).

The low molecular weight lipopeptide compounds produced by microorganisms are interesting class of amphiphilic membrane active biosurfactants. Among these cyclic lipopeptides, surfactins, iturins and fengycins produced by various *Bacilli* sp. have been characterized for their surface active properties, emulsifying activity, foaming property, hemolytic activity (Razafindralambo et al. 1998; Peypoux et al. 1999; Deleu et al. 1999) as well as for their potent antimicrobial activities (Winkelmann et al. 1983; Vanittanakom et al. 1986; Vater et al. 2002; Sing and Cameotra 2004). Surfactins are heptacyclic depsipeptides comprising two acidic amino acids (Glu^1^ and Asp^5^), four hydrophobic amino acids (Ile/leu^2,3,4,7^ and or Val^4^) and C_13–17_ β-hydroxy fatty acids (Peypoux et al. 1999; Vater et al. 2002). The Asp and Glu represent a minor polar domain which may stabilize divalent cations (Ca^+2^ a best fitting cation) required for the organization of micellar assembly (Heerklotz et al. 2004). The C_13–17_ β-hydroxy fatty acids along with Val/Leu/Ile amino acids contribute to a larger hydrophobic domain which interacts with acyl chain of hydrophobic substrate (hydrocarbons, oils) (Grau et al. 1999; Heerklotz et al. 2004). Iturin A is a cycloheptapeptide with seven amino acids (Asn^1,3,6^, Tyr^2^, Gln^4^, Pro^5^, Ser^7^) and C_13–16_ β-amino fatty acids, while fengycins are cyclic deca-depsipeptides with C_14–21_ β-hydroxy fatty acids and ten amino acids (Glu^1,5^, Orn^2^, Tyr^3,9^, Thr^4^, Ala/Val/Ile/Leu^6^, Pro^7^, Gln/Glu^8^, Val/Ile/Leu^10^) (Pathak et al. 2012). The surfactins have been characterized to be better surface active agents in comparison to iturins and fengycins. The fengycins are 40 and 70 times less hemolytic as compared to surfactins and iturins, respectively. When surfactin is present together with iturin A, it acts synergistically to lyse red blood corpuscles (Maget-Dana and Peypoux 1994; Thimon et al. 1995).

Banyan endophytic strain, *Bacillus subtilis* K1, produces heterogeneous mixture of 94 cyclic lipopeptide biosurfactants viz., seven surfactins, five iturins and 82 fengycins (Pathak et al. 2012). The surface tension of cell-free supernatant upon growth of *B. subtilis* K1 in Luria broth was found to be reduced to 27.9 mN/m. This prompted us to investigate the efficiency of crude cyclic lipopeptide extract obtained from fermentation broth of *B. subtilis* K1 in enhanced oil recovery. The present study describes surface active properties, stability of lipopeptide biosurfactant preparation and its suitability for enhanced oil recovery using laboratory scale sand pack column.

## Materials and methods

### Chemicals

All the chemicals used in this study were of analytical grade. Four stroke engine oil from Honda (SAE 20W40) was procured form local market.

### Medium

An optimized glucose minimal salt medium with following constituents in g/L: glucose, 50; KNO_3_, 5.0; KH_2_PO_4_·2H_2_O, 1.0; K_2_HPO_4_·2H_2_O, 1.0; MgSO_4_·7H_2_O, 0.2; CaCl_2_·2H_2_O, 0.02) was used for production of biosurfactants. Medium was sterilized by autoclaving at 121 °C for 15 min.

### Profile of growth, surface tension as well as emulsifying activity of *B. subtilis* K1

The cells from a single colony of a *B. subtilis* K1 were inoculated in 50 mL sterile Luria broth (LB) in 250 mL Erlenmeyer flask and incubated at 30 °C for 12 h (OD 1.9–2.0) on orbital shaker (150 rpm) and used as an inoculum. The appropriate aliquot of inoculum was added to 100 mL of sterile glucose minimal salt medium in 250 mL Erlenmeyer flasks to get an initial OD_600nm_ ~0.05. The flasks were incubated on orbital shaker (150 rpm) at 30 °C for 120 h and at regular interval of 24 h, one flask was removed, cells were separated by centrifugation (10,062×*g* for 20 min) and supernatant was monitored for surface tension and emulsifying activity. The cell pellet was used for measurement of growth by gravimetric method.

### Extraction of crude cyclic lipopeptides from fermentation broth

The cyclic lipopeptides from cell-free fermentation broth were precipitated by lowering its pH to 2 using 6 N HCl. The precipitates were harvested by centrifugation of acidified cell-free fermentation broth at 10,062×*g* for 20 min. The supernatant was discarded while the pellet was solubilized in pure methanol. The methanolic extract was then centrifuged to remove the undissolved fraction while supernatant was collected and subjected to rotary vacuum evaporation (Buchi, Switzerland) at 30 °C. The yellowish brown sticky substance thus obtained was used as cyclic lipopeptide preparation (CLP).

### Surface tension measurement

The surface tension (ST) was measured by DCAT-11 digital surface-tensiometer (Dataphysics, instruments, Filderstadt, Germany) using Wilhelmy plate, as described by Vater et al. (2002). All the measurements were made in triplicate and arithmetic averages and standard errors were calculated.

### Emulsifying activity, stability and emulsification index (E_24_)

The emulsifying activity (E.A) of culture supernatant as well as CLP was determined by using modified emulsification assay described by Navonvenezia et al. 1995. The 1 mL aliquot of culture supernatant was added to 6.5 mL of 20 mM TM buffer (20 mM Tris–HCl buffer [pH-7], 10 mM MgSO_4_) followed by addition of 0.1 mL of 1:1 (v/v) mixture of 2-methyl naphthalene and hexadecane. The samples were mixed vigorously for 2 min and allowed to stand for 1 h at 30 °C before measuring turbidity at 600 nm. One unit of emulsifying activity was defined as the amount of emulsifier that yielded an absorbance (600 nm) of 0.1 in the assay mixture. Similarly, the emulsifying activity of culture supernatant was also determined for tributyrin, groundnut oil, dodecane and olive oil. The emulsifying activity of some known chemical surfactants (Tween 20, Tween 40, Tween 60, Triton X 100 and SDS) was also determined by the same method. The hexadecane emulsions prepared using (0.1 mg%, w/v) CLP obtained from *B. subtilis* K1 as well as different chemical surfactants, were allowed to stand for 10 min at room temperature (30 °C ± 2) upon which absorbance at 600 nm was monitored after every 10-min interval up to 60 min. The log of the absorbance was plotted versus time and the slope of decay (decay constant, Kd) was expressed as the emulsion stability (Lee et al. 2006). For emulsification index (E_24_), 6 mL of hydrocarbon (hexane, heptane, octane or 4 stroke engine oil from Honda) was mixed with 4 mL of 48-h-old culture supernatant, vigorously for 5 min. The emulsion thus formed was allowed to stand for 24 h and emulsification index was determined by the following formula:

### Determination of critical micelle concentration (CMC)

The stock solution of CLP (10 mg/mL) dissolved in 10 mM Tris.Cl (pH-8) was prepared. The ST was measured using auto mode of DCAT-11 digital surface-tensiometer at 25 °C by automatic dilution of crude lipopeptide solution with distilled water till further increase in surface tension ceased. The surface tension was plotted against the Log concentration of biosurfactant using Origin 9.0 (OriginLab CO., MA, USA). The CMC was determined by measuring the intercept at two crossing lines.

### Stability of CLP

The stability of CLP at 100 °C, over a pH range from 2.0 to 12.0 and salinity (5–15 %, w/v NaCl) was investigated. The temperature stability was determined by monitoring ST of CLP before and after incubation at 100 °C for 2 h (allowed to cool to ambient temperature before monitoring ST). For pH stability, the pH of CLP was adjusted to pH 2.0, 4.0, 6.0, 8.0, 10.0 and 12.0 using 1 N HCl or 1 N NaOH and incubated for 2 days at 30 °C and then monitored for surface tension. The surface tension of CLP was monitored before and after incubation in solutions with varying salinity (5, 10 and 15 % (w/v) NaCl) for 2 days in order to determine its salt stability.

### Enhanced oil recovery using sand pack column

Sand pack column preparation and oil recovery experiment were carried out with minor modification in the sand pack method described by Suthar et al. (2008). A glass column (25 × 500 mm) was packed with 100 g of acid washed sand (100 mesh size). The brine solution (5 % NaCl, w/v) was then passed through the column and pore volume (PV) was determined by measuring the volume required to make the sand matrix wet in brine solution. To ensure 100 % saturation, three PVs of brine were passed through the column. After saturating column with brine solution, four stroke engine oil (Honda) was passed through the column under pressure until the column got saturated with oil. Once oil entered in the column, discharge of brine solution was observed from the matrix of sand. The discharged volume of brine from sand pack column was collected and measured to calculate initial oil saturation (Soi). The oil saturated column was washed with 4–6 PV of brine solution until no further oil was discharged in the effluent. The oil retained i.e. residual oil saturation (Sor) after brine solution wash was calculated on the basis of oil loaded and oil discharged in the effluent from the column. The 0.6 PV of 48-h-old cell-free fermentation broth containing lipopeptide biosurfactants was then loaded on to the oil saturated sand pack column and allowed to stand for 24 h. The amount of oil recovered upon incubation for 24 h was measured by collecting effluent in 10 mL fractions. This experiment was repeated thrice to evaluate the reproducibility and efficiency of *B. subtilis* K1 crude lipopeptide biosurfactant enhanced oil recovery using sand pack column. The percentage oil recovery was also calculated as described by Suthar et al. (2008).

## Results and discussion

### Profile of growth, surface tension and emulsifying activity of *B. subtilis* K1

**Fig. 1:**
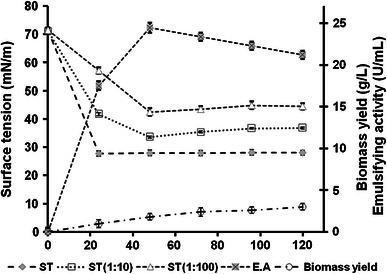
Profile of surface tension (ST) and emulsifying activity (EA) of cell-free fermentation broth as well as growth of *B. subtilis* K1

The surface tension and emulsifying activity of *B. subtilis* K1 culture supernatant were monitored along with growth during batch fermentation. The surface tension of the culture supernatant was found to reduce from 71.3 to 27.8 mN/m within 24 h of incubation and remained constant thereafter up to 120 h of fermentation (Fig. 1). Since surface tension of a solution is expected to remain constant for all surfactant concentrations above their critical micelle concentration, ST of diluted culture supernatant in distilled water was monitored as well. The ST values of 10- as well as 100-fold diluted culture supernatant decreased up to 48 h of incubation and remained constant thereafter suggesting that *B. subtilis* K1 produced and secreted biosurfactant up to 48 h in batch culture on glucose minimal salt medium. The emulsifying activity of culture supernatant increased along with accumulation of biosurfactant up to 48 h, in batch culture of *B. subtilis* K1, upon which it started decreasing with further incubation period (Fig. 1). The accumulation of biosurfactants in media during the early exponential phase up to mid- or late-logarithmic growth phase has been reported for several strains of *Bacillus* sp. and *Pseudomonas* sp. (Lee et al. 2006; Yakimov et al. 1997; Ghojavand et al. 2008; Joshi et al. 2008).

### Emulsification index and emulsion stability

**Fig. 2:**
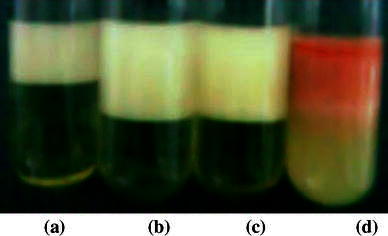
Emulsification of **a** hexane, **b** heptane, **c** octane and **d** crude engine oil using 48-h-old cell-free fermentation broth of *B. subtilis* K1

The culture supernatant of *B. subtilis* K1 was used to prepare emulsion of hexane, heptane and octane, which were found to remain stable up to 2 days while emulsion formed with four stroke engine oil (Honda) remained stable for more than 1 year (Table 1; Fig. 2). The emulsification index of culture supernatant was found to be least with hexane (33.3 %) and maximum with four stroke engine oil (93.3 %). Furthermore, it can be seen in Table 1 that emulsification index increased with increase in chain length of hydrocarbons. The typical engine oil is composed of hydrocarbons with chain length of 18–34 carbon atoms per molecule. The biosurfactants interact with hydrocarbons and form emulsion with aqueous medium. The hydrocarbons with higher alkyl chain length possess higher viscosity and to emulsify viscous hydrocarbon oil, high strength surfactant is required. The *B. subtilis* K1 produces a total of 94 variants of lipopeptide biosurfactants including 5 C_13–16_ iturinAs, 7 C_13–17_ surfactins and 82 C_14–21_ fengycins (Pathak et al. 2012). The amphiphilic nature of these cyclic lipopeptides imparts reduction in surface tension at oil water interface and stability of lipopeptide-oil emulsion foam (Razafindralambo et al. 1998). Iturins form mixed micelles with surfactins and exhibit synergism in lowering surface tension, foam formation and stabilization of liquid in foam (Razafindralambo et al. 1997). Similar synergism amongst variants of cyclic lipopeptides (surfactins, iturins and fengycins), present in biosurfactant preparation from *B. subtilis* K1, may be attributed toward formation of highly stable emulsion of four stroke engine oil. The surface active property and emulsifying activity of biosurfactants produced by *B. subtilis* K1 along with their stability toward extreme conditions suggest their application in enhanced oil recovery.

**Table 1:** Emulsification index (E_24_ (%)) and emulsion stability of biosurfactant produced by *B. subtilis* K1

Hydrocarbons	Hexane	Heptane	Octane	Honda engine oil
E_24_ (%)	33.3 ± 0.5	40.0 ± 0.6	50.0 ± 0.5	93.3 ± 0.7
Stability (days)	2	2	2	>1 year

Abu-Ruwaida et al. (1991) reported that emulsification index of surfactant produced by *Rhodococcus* ST-5 was lower for short chain hydrocarbons. The emulsification index values of *B. subtilis* K1 culture supernatant for hexane and heptane were found to be lower than those reported for *B. subtilis* LB5a (66.6 % for hexane and heptanes) (Nitschke and Pastore 2006). The higher emulsification index of *B. subtilis* K1 culture supernatant with four stroke oil suggests its application in enhanced oil recovery.

The emulsifying activity of crude biosurfactant from *B. subtilis* K1 was found to be highest with hexadecane and decreased with decrease in hydrocarbon chain length (Table 2). Significantly higher emulsification activity was observed with olive oil, indicating its application in preparation of olive oil-based cosmetics. Furthermore, the emulsification activity of biosurfactant from *B. subtilis* K1 was slightly better than Tween 20, Tween 40, Tween 60 and Triton X 100 except for the SDS (Table 3). The stability of hexadecane emulsion prepared using CLP from *B. subtilis* K1 was similar to SDS whereas it was lower in comparison to stability of emulsion formed employing non-ionic surfactants used in this study.

**Table 2:** Emulsifying activity of various substrates by crude biosurfactant preparation from *B. subtilis* K1

Substrate	Emulsification activity	
	(OD_600nm_)	(U/mL)
Hexadecane	0.9	9.0
Dodecane	0.8	8.4
Olive oil	0.8	8.1
Groundnut oil	0.3	3.2
Tributyrin oil	0.1	1.1

**Table 3:** Comparison of emulsifying activity of CLP from *B. subtilis* K1 with commercially available synthetic surfactants using hexadecane as substrate

Surfactant (0.1 mg/100 mL)	Emulsifying activity		Decay constant (Kd 10^−3^)
	(OD_600nm_)	(U/mL)	
Tween 20	0.88	8.8	1.1
Tween 40	0.89	8.9	0.6
Tween 60	0.79	7.9	0.7
Triton X-100	0.70	7.0	−0.8
SDS	1.1	10.1	−2.2
Biosurfactant	0.90	9.0	−2.2

### Critical micelle concentration (CMC)

**Fig. 3:**
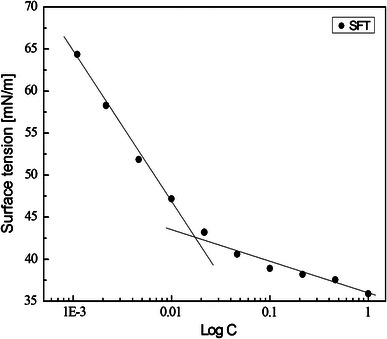
Surface tension (ST) of various concentrations of CLP from *B. subtilis* K1

The critical micelle concentration (CMC) of crude lipopeptide biosurfactant produced by *B. subtilis* K1 was found to be 17.5 μg/mL (Fig. 3). The CMC value for the CLP biosurfactant from *B. subtilis* K1 is significantly lower than CMC of acid precipitated biosurfactant (100 μg/mL) extracted from cell-free fermentation broth of *B. subtilis* group PTCC1696 (Ghojavand et al. 2008) as well as 1.7-fold (30 μg/mL) lower than crude lipopeptide biosurfactant produced by *B. subtilis* A8-8 (Lee et al. 2006). The CMC of CLP biosurfactant described in this study is also lower to the CMC of pure lichenysin B (20 μg/mL) obtained from *B. licheniformis* JF-2 (Jenneman et al. 1984). This suggests that CLP biosurfactant from *B. subtilis* K1 possesses a powerful surface tension lowering property.

### Temperature, pH and salinity stability of lipopeptide biosurfactant produced by *B. subtilis* K1

**Fig. 4:**
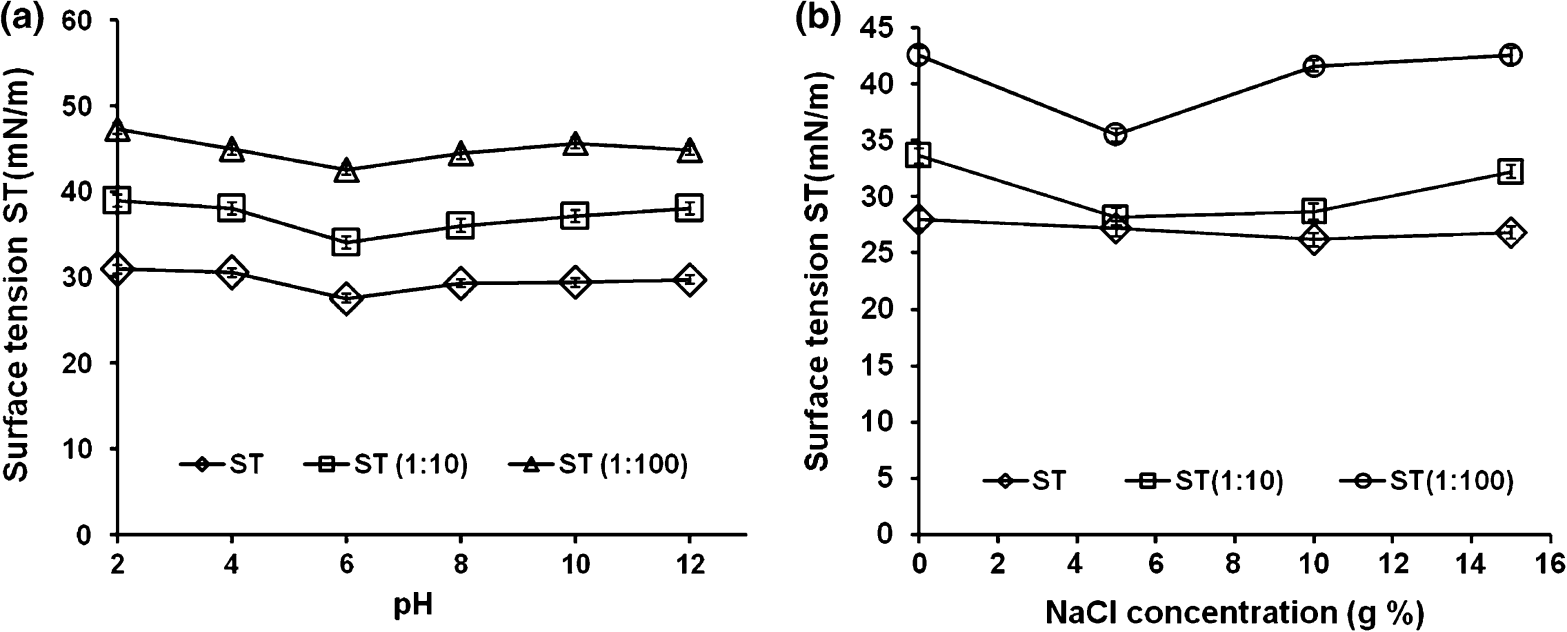
Effect of **a** pH, **b** salinity on biosurfactant activity of 48-h-old cell-free fermentation broth of *B. subtilis* K1 over a period of 2 days

The ST of culture supernatant remained constant even after incubation for 2 h at 100 °C, indicating its high thermostability (Table 4). The ST of culture supernatant also remained unaltered over a pH range of 6–12 even upon incubation for 2 days (Fig. 4a). The ST of *B. subtilis* K1 culture supernatant in the absence of NaCl was 27.6 mN/m and remained almost stable over a NaCl concentration up to 10 % (w/v) (Fig. 4b).

**Table 4:** Thermostability of biosurfactant produced by *B. subtilis* K1 at 100 °C for 2 h

Temperature (°C)	ST (mN/m)	ST (1:10) (mN/m)	ST (1:100) (mN/m)
100	27.6 ± 0.03	34.0 ± 0.03	42.5 ± 0.03
30	27.9 ± 0.03	33.6 ± 0.03	42.5 ± 0.03

Similar properties of stability at high temperature, over a wide pH range and salinity have been reported for surfactants produced by several *Bacilli* sp. (Vater et al. 2002; Nitschke and Pastore 2006; Ghojavand et al. 2008; Joshi et al. 2008).

### Enhanced oil recovery using CLP from *B. subtilis* K1

To evaluate the efficiency of biosurfactants produced by *B. subtilis* K1 in oil recovery process, sand pack column as a laboratory scale technique was employed. Table 5 shows the values for the various parameters viz., PV, OOIP, Sorwf, Sorbf, Swi(%), Soi(%), Sor(%), AOR(%) monitored for the determination of oil recovery, repeated thrice.

**Table 5:** Parameters for oil recovery using water flood and lipopeptide biosurfactant from *B. subtilis* K1 in sand pack glass columns

Parameters	Sand pack column (SPC)			SPC mean ± SE
	SPC-1	SPC-2	SPC-3	
PV (mL)	50.0	51.0	49.0	50.0 ± 0.6
OOIP (mL)	40.0	40.0	38.0	39.3 ± 0.7
Sorwf (mL)	20.0	20.0	20.0	20.0 ± 0.0
Sorbf (mL)	9.0	8.5	8.5	8.7 ± 0.2
Swi (%)	20.0	21.6	22.4	21.3 ± 0.7
Soi (%)	80.0	78.4	77.6	78.7 ± 0.7
Sor (%)	50.0	50.0	47.4	49.1 ± 0.8
Orecwf (%)	50.0	50.0	52.6	50.0 ± 0.9
OR (wf + bf) (%)	71.2	71.3	71.3	71.6 ± 0.4
AOR (%)	45.0	43.0	43.0	43.3 ± 0.9

*PV* Pore volume, *OOIP* Original oil in place (Amount of brine solution discharged upon displacement by oil sand pack column); Sowf—Oil retained after brine flooding; Sorbf—Oil released from sand pack column after treatment with biosurfactant; Soi  %—Percent initial oil saturation; Swi  %—Percent initial water saturation; Sor  %—Percent residual oil saturation; Orecwf  %—Percent oil recovery after water flooding; ORwf + bf  %—Percent oil recovery after water and biosurfactant flooding; AOR  %—Percent additional oil recovery after biosurfactant flooding (Suthar et al. 2008)

The value for PV, OOIP and Sorwf observed for the three replicates varied from 49 to 51, 38 to 43 and 14 to 20 mL, respectively. Upon flooding of column with brine, 32.50–52.63 % oil was recovered from the oil saturated column. This brine flooding step can be considered analogous to the water flooding operation in oil well during secondary phase oil recovery (Suthar et al. 2008). The remaining 47.4–67.5 % of oil in column was trapped in the sand matrix. The trapping of oil in sand pack column mimics the situation in oil well, where the oil gets trapped in the form of oil ganglia in pores of rocks (Shah 1981). To recover the oil trapped in the sand pack column, 0.6 pore volume of 48-h-old cell-free fermentation broth of *B. subtilis* K1 as a source of biosurfactant was loaded onto the oil saturated sand column. Upon 24 h of incubation, the oil displaced from the sand matrix was recovered and percentage additional oil recovery (AOR) was calculated. By using cell-free fermentation broth of *B. subtilis* K1, 43.3 ± 0.9 % of AOR was achieved. This AOR obtained in this study was comparable with the recovery achieved by the crude biosurfactant (43.1 ± 3.3 %) produced by *B. licheniformis* K125 (Suthar et al. 2008). The strain *B. mojavensis* JF2 isolated from the water injection brine of water-flood operation in Carter County, Oklahoma oil reserve has been reported for efficient oil recovery in core flood studies as well as from actual oil reservoirs (McInerney et al. 1999; Maudgalya 2005). *B. mojavensis* JF2 was used as standard strain for evaluation of MEOR by Suthar et al. (2008), and with this strain, 29.4 ± 5.6 % additional oil recovery over Sorwf was achieved. The AOR value obtained by cell-free fermentation broth of *B. subtilis* K1 was 13.9 % higher than the AOR obtained using culture supernatant of *B. mojavensis* JF2. Several strains of *Bacillus* sp. have been studied for their biosurfactants (Vater et al. 2002; Nitschke and Patore 2006; Ghojavand et al. 2008) and their application in enhanced oil recovery (Makkar and Cameotra 1998; Joshi et al. 2008; Suthar et al. 2008). The biosurfactants produced by *B. subtilis* MTCC 1427 and *B. subtilis* MTCC 2423 were shown to recover 56 and 62 % of oil from sand pack columns (Makkar and Cameotra 1998) which is significantly higher than that described in the present study. However, the CLP biosurfactant formed extraordinarily stable emulsion with four stroke engine oil, a property not reported so far in the literature. The biosurfactant from *B. subtilis* 20B was demonstrated to recover 30.2 % oil from sand pack column (Joshi et al. 2008) which is lower than that described in the present study. Thus, the crude biosurfactant obtained from culture supernatant of *B. subtilis* K1 seems to be a better agent for enhanced oil recovery from oil reservoirs.

## Summary and conclusion

The crude lipopeptide biosurfactant produced by *B. subtilis* K1 is a mixture of around 94 cyclic lipopeptides belonging to five surfactins, seven iturins and 82 fengycins and moreover these biosurfactants showed good emulsification activity, high stability and significantly good surface active properties. The crude lipopeptide biosurfactant was found to be stable over a wide range of pH, high temperature and high salinity, thus making it suitable for various industrial applications. The application of crude lipopeptide biosurfactant preparation in enhanced oil recovery using sand pack column in the laboratory was successfully demonstrated with 43.3 % oil recovery.
